# Hedgerows increase the diversity and modify the composition of arbuscular mycorrhizal fungi in Mediterranean agricultural landscapes

**DOI:** 10.1007/s00572-022-01090-5

**Published:** 2022-09-10

**Authors:** Guillermo González Fradejas, David García de León, Martti Vasar, Kadri Koorem, Martin Zobel, Maarja Öpik, Mari Moora, José María Rey Benayas

**Affiliations:** 1 Grupo de Ecología y Restauración Forestal (FORECO), Departamento de Ciencias de La Vida, Universidad de Alcalá, Alcalá de Henares, Spain; 2 Institute of Ecology and Earth Sciences, University of Tartu, Tartu, Estonia; 3 Fundación Internacional para la Restauración de Ecosistemas, Madrid, Spain

**Keywords:** AM fungi, Belowground biodiversity, Conservation ecology, Horticultural crops, Farmland

## Abstract

Sustainable agriculture is essential to address global challenges such as climate change and biodiversity loss. Hedgerows enhance aboveground biodiversity and provide ecosystem services, but little is known about their impact on soil biota. Arbuscular mycorrhizal (AM) fungi are one of the key components of belowground biodiversity. We compared the diversity and composition of AM fungal communities at four farmland sites located in Central Spain, where 132 soil samples in total were collected to assess soil physical and chemical properties and the AM fungal communities. We compared the richness (number of AM fungal taxa), taxonomic, functional, and phylogenetic diversity, and structure of the AM fungal communities across three farmland habitat types, namely hedgerows, woody crops (olive groves and vineyard), and herbaceous crops (barley, sunflower, and wheat). Our results showed positive effects of hedgerows on most diversity metrics. Almost 60% of the AM fungal taxa were shared among the three farmland habitat types. Hedgerows increased AM fungal taxonomic richness (31%) and alpha diversity (25%), and especially so compared to herbaceous crops (45% and 28%, respectively). Hedgerows harbored elevated proportions of AM fungi with non-ruderal life-history strategies. AM fungal communities were more similar between hedgerows and woody crops than between hedgerows and adjacent herbaceous crops, possibly because of differences in tillage and fertilization. Unexpectedly, hedgerows reduced phylogenetic diversity, which might be related to more selective associations of AM fungi with woody plants than with herbaceous crops. Overall, the results suggest that planting hedgerows contributes to maintain belowground diversity. Thus, European farmers should plant more hedgerows to attain the goals of the EU Biodiversity Strategy for 2030.

**Supplementary Information:**

The online version contains supplementary material available at 10.1007/s00572-022-01090-5.

## Introduction

Agriculture is a major source of environmental impact, contributing greatly to global biodiversity loss and climate change (IPCC 2022). The importance of implementing sustainable agricultural practices to address these challenges is highlighted in several EU strategies, such as the Biodiversity Strategy for 2030 and the Farm to Fork Strategy (European Commission 2020). In agricultural landscapes, hedgerows, which are linear structures of woody vegetation that surround agricultural fields (Lajos et al. 2020), can contribute to mitigate negative impacts (García de León et al. 2021). Hedgerows have been part of the traditional agricultural landscapes to separate different fields in many regions, but their occurrence and extent have significantly diminished because of agricultural intensification in Europe from the 1960s to the 1990s (van den Berge et al. 2021). Since the late 1990s, hedgerows have been partially restored (Staley et al. 2012).

Hedgerows have been used to maintain biodiversity and provide ecosystem services in agricultural landscapes (van Vooren et al. 2017; García de León et al. 2021). Aboveground biodiversity in hedgerows is higher than in cultivated areas, but lower than in natural areas such as forests (Slade et al. 2013). Hedgerows increase pollination (Morandin and Kremen 2013) and pest regulation (Morandin et al. 2014) and function as refugia of biodiversity (Rey Benayas and Bullock 2015). Thereby, hedgerows help to maintain the connectivity of agricultural landscapes (Rey Benayas and Bullock 2015), by being habitat or part of the habitat of associated plant, invertebrate and vertebrate species (Burgess et al. 2015; Mestre et al. 2018; Fialho et al. 2019). In addition, hedgerows improve water retention and infiltration (Holden et al. 2019) and counter erosion, compaction, and runoff (Monokrousos et al. 2006), thereby sustaining the overall quality of arable soils.

Despite the abovementioned benefits, the effects of hedgerows on soil biodiversity are not well studied. Soils are extremely biodiverse habitats, containing a wide range of fungi, bacteria, protists, and edaphic fauna. Soil biodiversity is directly linked to aboveground biodiversity (van der Putten et al. 2001; Wardle et al. 2004), affecting the functioning of terrestrial ecosystems (Bardgett and van der Putten 2014). One of the key organisms in soils are arbuscular mycorrhizal (AM) fungi, phylum Glomeromycota (Tedersoo et al. 2018), which colonize the roots of most (ca. 80%) vascular plant species (Smith and Read 2008). In this symbiosis, the host plant provides plant-assimilated carbon compounds to the fungi and profits in return from increased nutrient uptake (Smith and Read 2008) and resistance against abiotic and biotic stress, such as drought (Bitterlich et al. 2018) and pathogens (Akhtar and Siddiqui 2008). AM fungi associate with most woody and herbaceous crop plants (Bueno et al. 2021), being essential for ensuring sustainable food production for a growing population under a changing climate (de Vries and Wallenstein 2017).

The diversity and composition of AM fungi can be related in a bidirectional way to multiple factors, such as the dominant growth form of the plant species in a community (Sepp et al. 2018, 2021), or host plant functional groups (Davison et al. 2020). They also can be influenced by the levels of anthropogenic disturbance (García de León et al. 2018a). In agricultural landscapes, land use intensity has been shown to decrease AM fungal richness (Oehl et al. 2003), and diversity (Vahter et al. 2022). Management can alter the structure of AM fungal communities; intensively managed habitats harbor higher abundances of AM fungi with a ruderal life-history strategy compared to natural habitats (García de León et al. 2018a, b). Hedgerows can host species-rich AM fungal communities which resemble those of natural habitats and differ from the species-pool of AM fungal community characteristic of arable crops (Holden et al. 2019). Therefore, planting hedgerows has the potential to partially mitigate the negative effects of human disturbance on AM fungi in farmland.

The main goal of this study is to evaluate the effect of hedgerows on the diversity and structure of AM fungal communities in contrasting farmland habitat types. We hypothesized that (i) the richness and diversity of AM fungal communities are higher beneath hedgerows than in adjacent agricultural habitats and (ii) the increase in diversity is because of a high proportion of AM fungi with a non-ruderal life-history strategy. We expect that our study will support the need to maintain hedgerows in agricultural landscapes worldwide and, specifically, European strategies intended to increase biodiversity in farmland.

## Material and methods

### Study sites and sampling

Four sites where the International Foundation for Ecological Restoration (https://fundacionfire.org/) planted hedgerows between 2009 and 2015 were selected for this study. The sites were in the Toledo and Ciudad Real provinces, Central Spain, and had well-developed hedgerows in 2020, when our field sampling took place (Fig. S1a). The hedgerows were composed of shrubby woody plants characteristic of the Mediterranean climate such as broom (*Retama sphaerocarpa*), hawthorn (*Crataegus monogyna*), rose (*Rosa canina*), bladder-senna (*Colutea arborescens*), jasmine (*Jasminus fruticans*), buckthorn (*Rhamnus alaternus* and *Rhamnus lycioides*), and others. The hedgerows surrounded three olive groves, namely “El Peral” (OD; Fig. S1b; 38°48′N, 3°21′W; 1.76 hectares), “Vista Alegre” (OV; Fig. S1c; 38°48′N, 3°′W; 4.55 hectares), “Fuente del Albañal” (ON, Fig. S1d; 40°3′N, 4° 17′W; 2.65 hectares), and a barley field at the time of sampling at the “Los Billares” site (CN; Fig. S1e; 40°1′N, 4°14′W; 12.66 hectares). The four sites did not exhibit large differences in hedgerow composition or plant richness, which was 13 species in “Fuente del Albañal,” 14 species in “El Peral” and “Vista Alegre” and 18 species in “Los Billares” (Table S1). Adjacent to the hedgerows, there were a barley field, a grassland, olive orchards, a sunflower field, vineyards, and a winter wheat field (Table 1). “El Peral” was managed as an organic farm, including mulching with spontaneous herbs. “Los Billares” followed the cropping sequence cereal-leguminous-fallow. “Fuente del Albañal” had been a vineyard and turned into an olive orchard at the time of hedgerow planting.

**Table 1:** Description of experimental design, year of hedgerow planting, land use history prior to hedgerow planting, and crops adjacent to hedgerows at every site

Site	Number of samples in hedgerows	Number of samples in herbaceous habitats	Number of samples in woody habitats	Year of hedgerow planting	Land-use history prior to hedgerow planting	Monocrops adjacent to the hedgerow
“Vista Alegre”	5	5	5	2009	Olive orchard	Olive orchard and sunflower
“El Peral”	11	0 (2)	17	2010	Olive orchard	Grasslands and olive orchard
“Fuente del Albañal”	7	2	9	2009	Vineyard, which turned into an olive orchard at the time of hedgerow plantation	Olive orchard, vineyard, and wheat
“Los Billares”	19	27	8	2011–2015	Winter cereal-leguminous crop-fallow	Barley, vineyard, and wheat

The two samples in brackets at “El Peral” represent a grassland and were excluded from analyses due to their low representativity (i.e., grasslands are never plowed, despite being herbaceous habitats)

The studied sites have a Mediterranean climate, with cold rainy winters and a long summer drought that imposes severe water stress to the vegetation. The mean annual temperatures and total annual rainfall averaged 15.47 °C and 393 mm for the Ciudad Real sites, and 15.67 °C and 457 mm for the Toledo sites in the period 2010–2016. Soils are deep and fertile cambisols from limestone in the Ciudad Real sites and luvisols from arkose in the Toledo sites. Descriptive values of soil geochemistry are reported in Table 2.

**Table 2:** Soil physical and chemical parameters of study sites (mean ± standard error)

Variable	Los Billares (20/11/2020)	El Peral (23/11/2020)	Fuente del Albañal (20/11/2020)	Vista Alegre (23/11/2020)
pH	7.85 ± 0.07 b	8.35 ± 0.04 a	8.27 ± 0.02 a	8.35 ± 0.04 a
%C	0.40 ± 0.02 b	0.84 ± 0.07 a	0.42 ± 0.03 b	0.89 ± 0.08 a
%OM_oxidable_	0.68 ± 0.04 b	1.41 ± 0.11 a	0.74 ± 0.04 b	1.43 ± 0.13 a
%OM_total_	0.89 ± 0.04 b	1.83 ± 0.14 a	1.00 ± 0.06 b	1.82 ± 0.17 a
%N	0.03 ± 0.01 a	0.05 ± 0.01 b	0.03 ± 0.01a	0.05 ± 0.01 b
C:N	17.12 ± 0.20 a	17.31 ± 0.24 a	17.25 ± 0.29 a	17.19 ± 0.33 a
P_2_0_5_	8.14 ± 0.41 a	8.51 ± 0.69 a	7.92 ± 0.88 a	7.49 ± 0.81 a

Different letters indicate significant differences within rows by Tukey post-hoc analyses with Bonferroni correction after Kruskal Wallis statistical tests (n = 115). OM stands for Organic Matter. The pH was potentiometrically measured in a 1:2.5 soil/water suspension

We took 132 soil samples in total at the four sites between the 20th and 23rd of November 2020. No root samples were taken, but only samples from the soils in which hedgerows and crops were growing. Sampling took placed every 50 m along the hedgerows, so that the number of sampling spots depended on field size. There were five, eleven, nine, and twenty sampling spots at the “Vista Alegre,” “El Peral,” “Fuente del Albañal,” and “Los Billares” sites, respectively. At each sampling spot, three samples were collected, one within the hedgerow and two others at 50 m to the left and to the right of the hedgerow, i.e., in the adjacent fields. The surrounding habitat was classified as woody or herbaceous crops. Woody crops were olive groves (*Olea europaea*) and vineyards (*Vitis vinifera*), both AM plant species, whereas the herbaceous crops were winter barley (*Hordeum vulgare*), sunflower (*Helianthus annus*), and winter wheat (*Triticum aestivum*). Three out the expected 135 samples could not be collected because of a ditch. Twelve samples did not pass the sequencing quality control, as they contained too little soil to extract sufficient DNA. Two samples represented grasslands and were removed from further analyses because of their low representativity. At each sampling point, a 20-cm-deep soil cylinder was extracted with a 5-cm-diameter auger for soil physical and chemical analyses (around 20 g of fresh soil) and for DNA metabarcoding (5 g). After excluding samples with fewer than 100 AM fungal reads and singletons (see “Bioinformatics”), all analyses were made on soil samples from single soil cores (*n* = 115).

### Laboratory analyses

Soil for physical and chemical analyses was dried at room temperature to constant weight and carefully sieved through a 2-mm mesh. The coarse material was discarded, and the remaining fine-ground fraction was homogenized prior to the analyses. pH, oxidable organic matter (i.e., labile carbon), total organic matter, the organic carbon, phosphorus, and nitrogen concentrations were determined following the methods described in Peech et al. (1947), Nelson and Sommers (1983), Olsen et al. (1954), and Bremner and Mulvaney (1982), respectively (Table 2).

The samples collected for AM fungal metabarcoding were preserved in silica gel until April 2021 and then sent to Macrogen Inc. (https://www.macrogen.com/en/main) to extract, PCR amplify, and sequence DNA. DNA was extracted as recommended in Lekberg et al. (2018), using the DNeasy^®^ PowerMax^®^ Soil Kit (Qiagen) Nextera XT preparation kit (index PCR step), and amplified with the primers WANDA (5′-CAGCCGCGGTAATTCCAGCT-3′) and AML2 (5′-GAACCCAAACACTTTGGTTTCC-3′) (Lee et al. 2008; Dumbrell et al. 2011), targeting the 18S rDNA marker. The Nextera XT-indexed PCR amplicons were sequenced, using a 2 × 300 bp paired end read sequencing approach on a MiSeq Illumina sequencer with Reagent Kit v3 (600 cycles).

### Bioinformatics

Paired-end Illumina reads were cleaned using the gDAT pipeline (Vasar et al. 2021). In short, reads were demultiplexed into samples using an 8-bp barcode allowing one mismatch for both forward and reverse reads. Demultiplexed reads were checked for correct forward (WANDA) and reverse (AML2) primers allowing one mismatch for both primers. Both reads were selected if the average quality of the sequences was ≥ 30. Filtered paired-end reads were combined with FLASH (v1.2.11, Magoč and Salzberg 2011) using default parameters (overlap ≥ 10 bp, identity ≥ 75%). Chimeric sequences were removed with VSEARCH (v2.15, Rognes et al. 2016) using default parameters in reference database mode with the MaarjAM database (status September 2021, Öpik et al. 2010). The obtained reads were assigned to virtual taxa (v2.13, Öpik et al. 2010) using the MaarjAM database by conducting a BLAST + search (Camacho et al. 2009). From a BLAST + search for each sequence, the best hit was identified using 97% identity and 95% alignment thresholds. Reads that did not achieve a hit against the MaarjAM database (nohits) were subjected to a BLAST + search against the INSDC non-redundant nucleotide database (status September 2021, Karsch-Mizrachi et al. 2018), with lowered thresholds of 90% identity and 90% alignment to detect potential novel VT absent from the MaarjAM database. Nohits against INSDC were distributed as follows: fungi ~ 53% (Glomeromycota ~ 7%), metazoa ~ 33%, and plants ~ 6%. Three novel VT were added to a final BLAST + against the MaarjAM database. These novel VT were incorporated in the phylogenetic tree published by García de León et al. (2018a) to calculate the cophenetic distance for phylogenetic analyses. Two soil samples yielding fewer than 100 AM fungal reads and VT that were represented with one read (singletons) were omitted from further analysis, resulting in a total of 115 samples used for statistical analyses. Representative sequences were uploaded to the GenBank database with accession number KFUP0000000.

### Statistical analyses

Richness was evaluated as the number of virtual taxa per sample. A virtual taxon (VT) is a phylogenetically delimited cluster of the middle part of the SSU rRNA gene, as curated by the MaarjAM database (Öpik et al. 2010). Rarefaction methods use observed data to normalize VT abundances, based on the finite sample size (Chao et al. 2014; Hsieh et al. 2016). Rarefied richness to the median number of sequences and rarefied richness to the minimum number of sequences were calculated as indicators of relative VT abundance within individual soil samples using function *rarefy* from the *vegan* R package (Oksanen et al. 2022).

Alpha taxonomic diversity was computed with the *iNEXT* R package as the exponential Shannon index based on an asymptote to compensate for the differences (i.e., interpolate/extrapolate) in sequencing depth (Chao et al. 2014; Hsieh et al. 2016). The proportion of VT that have been cultivated in cultures has been proposed as a proxy of alpha functional diversity because cultured taxa represent a ruderal life-history strategy (Ohsowski et al. 2014; García de León et al. 2018a, b). Specifically, alpha functional diversity was calculated as the log (uncultured/(cultured + uncultured)) taxa. Alpha phylogenetic diversity was calculated following Tucker et al. (2017) as the phylogenetic richness (pd), the mean pairwise distance (mpd), and the mean nearest taxon distance (mntd) as implemented in the *cophenetic* function from the *ape* R package (Paradis and Schliep 2019).

Differences in the richness, diversity, and structure of AM fungal communities in soil of different farmland habitats (hedgerows versus woody and versus herbaceous crops) were tested using one-sample, one-sided Student’s *T* tests with the function *t.test* from the *stats* R package (R Core Team 2021). Adding a subset argument to stratify Student T tests by site did not produce any changes in results; thus, the simple unstratified model is presented. Differences in soil characteristics among sites were tested using Tukey post hoc analyses with Bonferroni correction after Kruskall Wallis tests (Table 2). To assess the strength of relationships between biodiversity metrics and soil characteristics, Kendall’s tau rank correlation coefficient was used with a Bonferrroni correction as implemented by the *cor.test* function from the *stats* R package (Table S2).

Nonmetric multidimensional scaling (NMDS) based on the Bray–Curtis distance was used to visualize similarity in the structure (i.e., relative VT abundance within individual soil samples) of AM fungal communities among habitat types, using the function *monoMDS* from the *vegan* R package (Oksanen et al. 2022). Permutation multivariate analysis of variance (PERMANOVA) with 9 999 replications was conducted using the function *adonis* in R package *vegan* to assess the effects of farmland habitat and site on AM fungal community structure (Table S3). Indicator taxon analysis was performed using function *indval* in the *labdsv* R package (Roberts 2019); we defined a threshold indicator value of 25% to identify good indicator taxa (Dufrêne and Legendre 1997). Data files are provided in the supplementary materials (Data S1–S5).

## Results

### Soil characteristics

Soil characteristics differed among sites (Table 2) although differences in soil geochemistry were small among habitat types (results not shown). Site “Los Billares” showed a significantly lower pH than any of the olive groves. Sites in the Toledo province (“Los Billares” and “Fuente del Albañal”) had less soil carbon, oxidable organic matter, total organic matter, and nitrogen than sites in the Ciudad Real province (“Vista Alegre” and “El Peral”). The ratio C:N and phosphorus were similar in Toledo and Ciudad Real sites. Soil reaction (pH) was positively correlated with richness and negatively with phylogenetic divergence (Table S3). Carbon content, oxidable organic matter, total organic matter, and nitrogen concentration were positively correlated with AM fungal richness. Carbon content, oxidable organic matter, and total organic matter were negatively correlated with phylogenetic divergence calculated as the mean nearest taxon distance.

### Biodiversity metrics

**Fig. 1:**
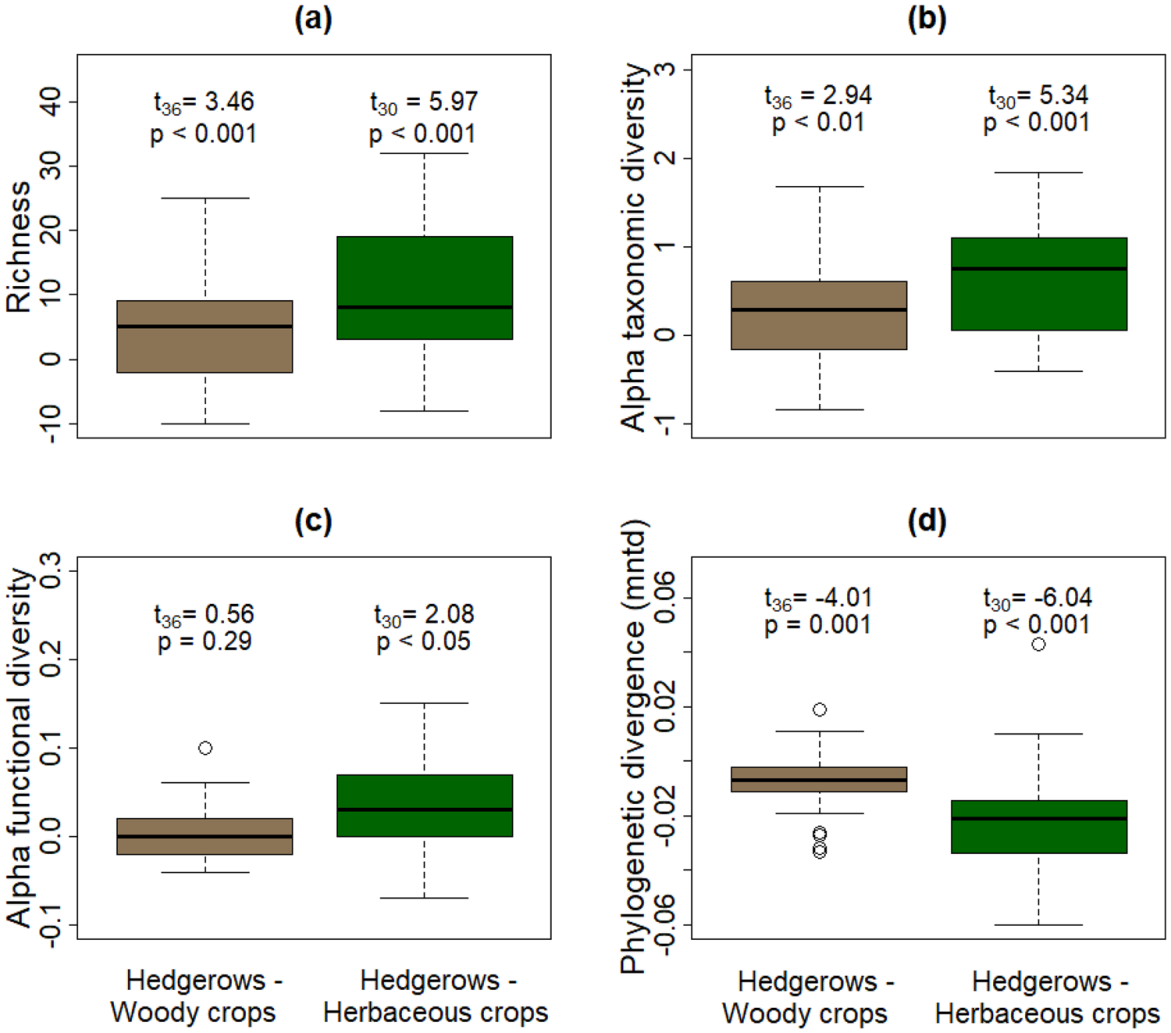
Richness (i.e., number of virtual taxa (VT), **a** taxonomic alpha VT diversity (i.e., Shannon diversity index, **b** functional VT diversity (i.e., proportion between uncultured and all taxa, **c** phylogenetic VT divergence (i.e., mean nearest taxon distance), **d** of arbuscular mycorrhizal fungi by farmland habitat type. Ordinates show the differences (not the actual values) between hedgerow and crop samples in each sampling spot (the hedgerow sample arbuscular mycorrhizal fungal biodiversity metric in sampling spot X minus the crop sample arbuscular mycorrhizal fungal biodiversity metric in sampling spot X). These differences were tested based on one-tailed Student *T* tests for positive effects of hedgerows. The thick black horizontal line displays the median difference of a given biodiversity metric. Boxes are constrained by interquartile range. Whiskers are limited by 1.5 times the interquartile range beyond the first and third quartiles. Open circles represent possible outliers. Text below boxes indicates the Student *t*-statistic value, subscripts show the degrees of freedom, and p represent the associated probability

The richness (Fig. 1a) and taxonomic diversity (Fig. 1b) of the AM fungi differed in farmland habitat types, being significantly higher in hedgerows than in woody or herbaceous crops. Rarefaction to the median (Fig. S2a) and to the minimum (Fig. S2b) number of sequences supported an increase of relative VT abundance in hedgerows. The functional diversity (i.e., the proportion of uncultured AM fungal taxa, Fig. 1c) and phylogenetic richness (Fig. S2c) were significantly higher in hedgerows than in herbaceous crops, but similar to those of woody crops. Hedgerows had lower phylogenetic divergence than herbaceous crops (Fig. 1d, Fig. S2d) and woody crops (Fig. 1d).

### Community structure

**Fig. 2:**
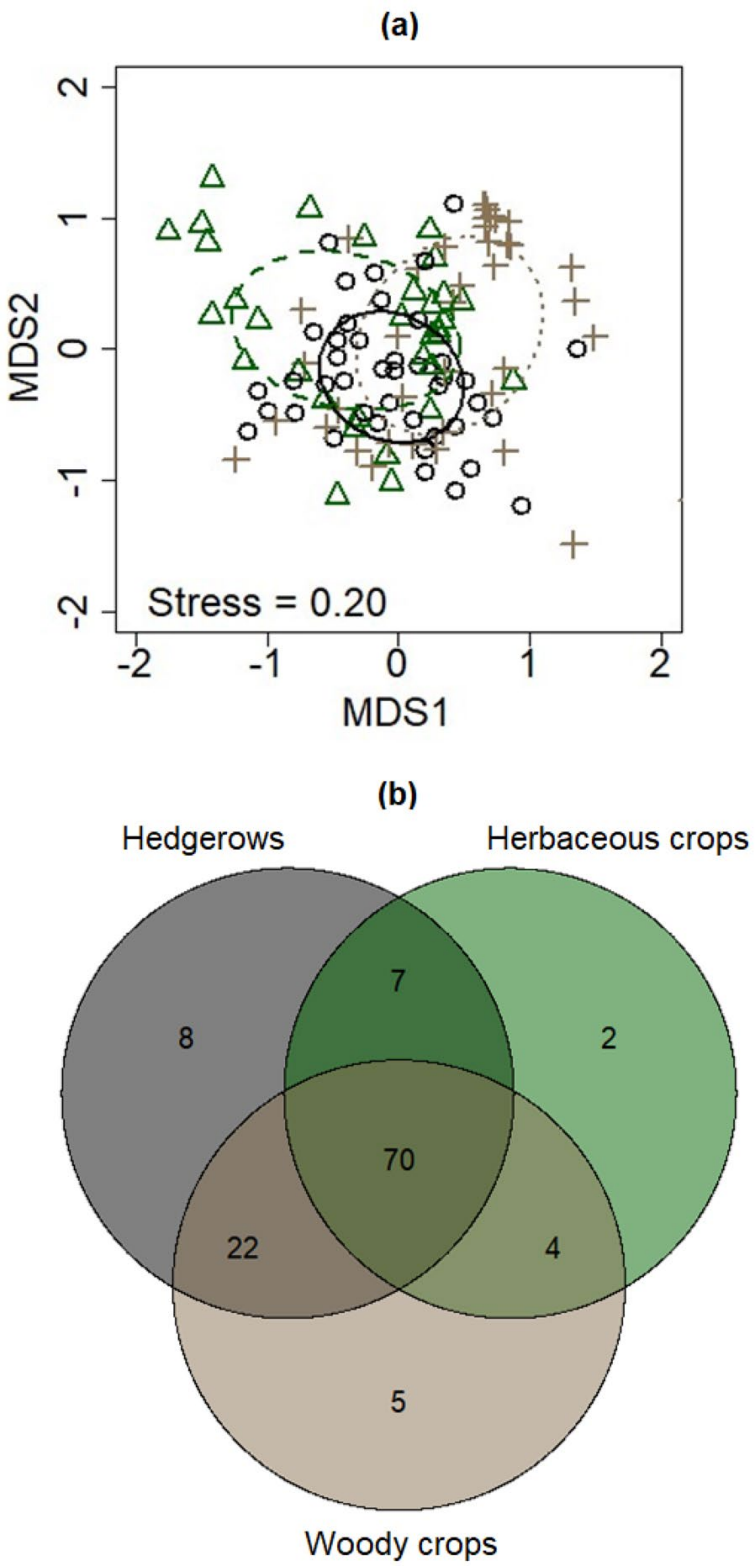
Non-metric multidimensional scaling ordination plot (*n* = 115) showing the structure of AM fungal communities (i.e., based on relative VT abundance, **a** and Venn diagram **b** for the three farmland habitat types. Black dots and solid ellipse indicate hedgerows. Green triangle and dashed ellipse display herbaceous crops. Brown crosses and dotted ellipse shows woody crops. The size of ellipse represents the standard deviation from the group centroid. The figures in the Venn diagram indicate the number of unique and shared virtual taxa among habitat types

AM fungal community structure showed significant differences among farmland habitat types (Fig. S2a, Table S3) and sites (Table S3). Seventy out of a total of 118 VT were found in all farmland habitat types (Fig. 2b), with the AM fungal community sharing more VT between hedgerows and woody crops than between hedgerows and herbaceous crops and between crops. There were two unique VT beneath herbaceous crops, both of which belonged to the *Glomus* genus. Hedgerows harbored eight unique VT, seven *Glomus*, and one *Acaulospora*. Woody crops hosted five unique VT, two *Glomus*, one *Scutellospora*, one *Racocetra*, and one *Diversispora* VT*.* Some of the unique VT in hedgerows were very common in agricultural landscapes according to the MaarjAM database. Indicator taxa analysis showed that hedgerows favor *Diversispora* and *Glomus* VT (Table S4). Woody crops favored two *Glomus* taxa (VT113 and VT387) and one *Claroideoglomus* taxon (VT357). Indicator species for herbaceous crops belonged to *Archaeospora*, *Pacispora*, and *Paraglomus* genera.

## Discussion

Overall, our results showed positive effects of hedgerows on the richness, alpha taxonomic, and functional diversity of AM fungi. Hedgerows also influenced community structure, harboring AM fungal communities more typical of woody than herbaceous crops. These findings suggest that hedgerows have potential to be used as a tool for maintaining soil biodiversity of agricultural landscapes, especially compared to herbaceous crops.

### Hedgerows enhance AM fungal diversity in farmland habitats

Hedgerows increased the richness and diversity of AM fungal communities in comparison with crops, supporting our first hypothesis. Hedgerows have been considered an intermediate state between natural woodlands and crops in terms of aboveground biodiversity (Slade et al. 2013; García de León et al. 2021). Our results suggest that they benefit belowground biodiversity in a similar way. Planting hedgerows is a form of revegetation in agroforestry. Revegetation previously has been shown to benefit the diversity of AM fungi in degraded environments such as old mines (Juge et al. 2021). As hedgerows are not subjected to agricultural practices such as tillage, harvesting, or fertilization, which lead to the reduction of AM fungal diversity (Oehl et al. 2003; Vahter et al. 2022), they can provide suitable habitat for a high number of fungus taxa. Moreover, we studied hedgerows with higher host plant richness than crop habitats (i.e., olive grove, vineyard, barley, sunflower, and wheat monocrops). Previous studies have shown a strong correlation between AM fungal and plant host richness (Martínez-García et al. 2015), suggesting that a change in richness of one of the partner types of the symbiosis may cause a change in the same direction in the other partner type (Hiiesalu et al. 2014; García de León et al. 2016a), thereby linking the changes in above- and belowground biodiversity.

Plowing frequency might help to explain the difference in taxonomic and functional diversities of AM fungi in our study system in that cereal fields are plowed yearly, while the studied olive groves are plowed only once every 5 years and hedgerows are never plowed. High frequency of plowing can destroy hyphal networks; Helgason et al. (1998) pointed to plowing as a leading cause for low diversity of AM fungi in arable fields. A recent study, however, has reported no significant effects of plowing (Vahter et al. 2022), suggesting that its impacts may be context-dependent.

Hedgerows and woody crops showed a high prevalence of uncultured or non-ruderal AM fungal taxa, supporting our second hypothesis. High prevalence of uncultured AM fungal taxa previously has been related to natural environments while the proportion of cultured (ruderal) taxa generally is higher in anthropogenic habitats (Ohsowski et al. 2014; García de León et al. 2018a). Ruderal AM fungal taxa possess traits such as a short life cycle and fast growth (Chagnon et al. 2013) that can make them resilient to disturbances and enable fast colonization of host plants, which may explain their dominance in frequently tilled agricultural soils. The observed high proportion of non-ruderal taxa in wooded habitats, and particularly in hedgerows, suggests that these habitats bear similarities to natural habitats. This result is particularly interesting because it contrasts with Bainard et al. (2013) who reported a lack of functional complementarity among the AM fungal taxa of tree-based intercrops and monocrops.

Opposite to findings by Manoharan et al. (2017), our results found that hedgerows harbored a significantly lower phylogenetic diversity (i.e., richness and divergence) of AM fungi than did herbaceous crops. As the number of samples under herbaceous crops were more prevalent in “Los Billares” than in any other site, the higher phylogenetic diversity under herbaceous crops might be related to differences among sites (Tucker et al. 2017). For instance, the distance to sources of AM fungal propagules might be longer than in the olive groves because of the large field size in “Los Billares.” García de León et al. (2016b) suggested that AM fungal spores have difficulty colonizing at distances longer than 40 m. Apparent dispersal limitation, however, alternatively might be an artifact of covariation between taxonomic richness and phylogenetic diversity (i.e., the lower the number of taxa, the further apart in a phylogenetic tree one can expect to find two taxa selected at random). Supplementary correlation analyses support strong relationships between taxonomic richness and phylogenetic diversity (pd: *τ* = 0.84, *t*_103_ = 15.88, *p* < 0.01; mpd: *τ* = − 0.45, *t*_103_ = − 5.06, *p* < 0.01; mntd, *τ* = − 0.74, *t*_103_ = − 1.12, *p* < 0.01).

Another alternative explanation to the observed pattern is that all possible AM fungal taxa arrive to the most disturbed habitats (i.e., herbaceous crops). But only a fraction of them will be able to compete and develop under less disturbed habitats. Woody habitats and, particularly, hedgerows might have acted as ecological filters (Fig. 2a). This interpretation is in line with Battie-Laclau et al. (2020) who found that AM fungal diversity in hedgerows differs from that at 11 m within adjacent crops because monocrops are fertilized, and hedgerows are not. Arguably, dispersal limitation is more likely than the ecological-filter hypothesis to explain the relatively low phylogenetic diversity observed in hedgerows. This may be because the spatial scale in our study [50 m] was closer to 40 m (García de León et al. 2016b) than to 11 m (Battie-Laclau et al. 2020).

### Community structure

The low phylogenetic diversity of AM fungi in hedgerows further suggests that farming activities do not lead to a clear selection pressure on AM fungal communities and that community structure is determined to some extent by stochastic processes (Dumbrell et al. 2010). These stochastic processes may favor AM fungal properties that are not phylogenetically conserved (e.g., ruderability). Hedgerows had AM fungal communities more like woody crops than to those of herbaceous crops, suggesting selective symbiotic associations. The presence of a woody host plant previously has been found to structure the composition of AM fungal communities at a global scale (Öpik et al. 2006; Davison et al. 2015) and at local scale (Moora et al. 2014; Sepp et al. 2021). Host functional group (Davison et al. 2020) and identity (Martínez-García et al. 2015) have been argued to be important factors in shaping AM fungal community composition. Future studies on the AM fungal communities in roots are needed to evaluate the role of host plant identity in structuring their composition and diversity (Varela-Cervero et al. 2015).

### Soil characteristics

“Los Billares” had a lower soil pH than any other site. Nitrogen fertilization is known to lead to soil acidification in agricultural fields (Zhang et al. 2016). Nitrogen concentration was positively related to the richness (Table S2). This explanation would be in line with the findings by Peyret-Guzzon et al. (2016) who concluded that physical soil disturbance and fertilization of a buffer strip cause shifts in the structure of AM fungal communities. However, nitrogen cannot explain the lowest pH found at “Los Billares” because its concentration in “Los Billares” was similar to that in “Fuente Albañal,” where pH was as basic as in the Ciudad Real sites (Table 2). Conversely, a higher frequency of tillage may have reduced the pH at the barley field (see discussion about tillage effect above).

High levels of soil carbon and organic matter were associated with high AM fungal richness. Overall, the observed relationships between AM fungi and soil characteristics support potential effects of AM fungi on soil organic matter dynamics. It is also possible that soil characteristics influence AM fungal diversity (Table S2). Previous studies have argued that AM fungi can increase carbon sequestration by increasing host plant photosynthesis and decreasing carbon release to the atmosphere through plant respiration (Wang et al. 2016). The positive relation between the richness of AM fungi and soil organic matter does not contravene such an hypothesis. However, such a statistically limited observational study as ours cannot be used to suggest with certainty that AM fungi determine soil organic carbon in farmland. Previous studies have argued that AM fungi can contribute to the accumulation of organic matter in the soil through their own dead tissues and that the hyphal network can facilitate the distribution of soil carbon throughout soil pores to places where it can be protected from mineralization (Frey 2019). Future work must increase sampling beyond that of this current observational study and should experimentally examine influence of AM fungi on the carbon cycle.

## Conclusions

Hedgerows support diverse AM fungal communities and can help maintain AM fungal diversity in Mediterranean agricultural landscapes, although their similarities with woody crops make them a most interesting tool in the case of herbaceous crops. As hedgerows experience little influence of agricultural practices and are a habitat involving a rich host plant community, they are associated with higher richness and diversity of AM fungi than horticultural and arable crops. Hedgerows are efficient in providing habitat to AM fungi, supporting the maintenance of landscapes with high soil biodiversity. They also are able to provide a better habitat for AM fungi with a non-ruderal life-history strategy than herbaceous crops, maintaining this functionality in agroecosystems.

## Supplementary Information

Below is the link to the electronic supplementary material.

Supplementary file1 (PDF 299 KB)

Supplementary file2 (PDF 466 KB)

Supplementary file3 (PDF 468 KB)

Supplementary file4 (PDF 378 KB)

Supplementary file5 (PDF 462 KB)

Supplementary file6 (PDF 508 KB)

Supplementary file7 (PDF 590 KB)

Supplementary file8 (XLSX 66.5 KB)

Supplementary file9 (XLSX 19.0 KB)

Supplementary file10 (XLSX 16.6 KB)

Supplementary file11 (XLSX 203 KB)

Supplementary file12 (TXT 3.29 MB)
