# Supplementary material for: Hedgerows increase the diversity and modify the composition of arbuscular mycorrhizal fungi in Mediterranean agricultural landscapes
Source: Mycorrhiza. 2022 Sep 10;32(5-6):397–407. doi: 10.1007/s00572-022-01090-5 (PMC9561024; doi:10.1007/s00572-022-01090-5)
Supplement: Supplementary file 1 — Supplementary file1 (PDF 299 KB) [file 572_2022_1090_MOESM1_ESM.pdf]

List of supplementary material

Table S1. Woody plant species in hedgerows by site. First column indicates woody plant species. Second to fifth columns are different sites.

Table S2 Correlations among soil parameters and biodiversity metrics. Values shown represent Kendall´s tau rank correlation coefficient (τ), with a Bonferroni correction. Subscripts show the degrees of freedom of T student tests against zero; p indicate the p-value. Significant values are highlighted in bold. Data for building this table excluded outliers. Biodiversity metrics are showed in order of apparition in main text. OM stands for organic matter.

Table S3. Complete PERMANOVA table. Habitat type codes for "Hedgerows", "Herbaceous crops", and "Woody crops". "Site" codes for "El Peral", "Vista Alegre", "Fuente del Albañal", and "Los Billares"; r indicates the Pearson correlation coefficient. F is the threshold statistic; p stands for p-value.

Table S4. Indicator taxa by farmland habitat type. Only indicator values higher than 0.25 are shown.

Figure S1. Situation map (a) and aerial photographs of the studied fields taken from SIGPAC (https://sigpac.mapama.gob.es/fega/visor/). "El Peral" (b; 38º48´N, 3º21´W; 1.76 hectares), "Vista Alegre" (c; 38º48´N, 3º 13´W; 4.55 hectares), "Fuente del Albañal" (d; 40º3´N, 4º 17`W; 2.65 hectares), and "Los Billares" (e; 40º 1´N, 4º 14´W; 12.66 hectares) sites.

Figure S2 Rarefied richness to the median number of VT sequences (a), rarefied richness to the minimum number of VT sequences (b), phylogenetic VT richness (i.e., phylogenetic diversity index, c) and phylogenetic VT divergence (i.e., mean pairwise distance, d). Ordinates show the differences (not the actual values) between hedgerow and crop samples in each sampling spot (the hedgerow sample arbuscular mycorrhizal fungal biodiversity metric in sampling spot X minus the crop sample arbuscular mycorrhizal fungal biodiversity metric in sampling spot X). These differences were tested based on one-tailed Student T tests for positive effects of hedgerows. The thick black horizontal line displays the median difference of a given biodiversity metric. Boxes are constrained by interquartile range. Whiskers are limited by 1.5 times the interquartile range beyond the first and third quartiles. Open circles represent possible outliers. Text below boxes indicates the Student *t*-statistic value, subscripts show the degrees of freedom, and p represent the associated probability.

Data S1 Raw data. This file is the output of the *gDAT* pipeline (Vasar et al. 2021) based on the collected samples. ´Sample/Hit´ codes for the collected samples. The first two letters represent the Site [´OD´ stands for "El Peral", ´OV´ means "Vista Alegre", ´ON´ is "Fuente del Albañal", ´CN´ refers to "Los Billares"]. The number in column ´Sample/Hit´ represents the sampling spot [1-20] within a site. Capital letters after the sampling spot codes for the position within the sampling spot [A = Fifty meters to the left of hedgerow, B = Within the hedgerow, C = Fifty meters to the right of hedgerow]. Column ´taxa count´ contains the taxonomic richness (i.e., number of virtual taxa) per sample. Column ´Cleaned reads´ provides the total number of reads per sample; Column ´Total´ shows the number of arbuscular mycorrhizal (AM) fungal sequences per sample. Row ´Samples count´ indicate in how many samples a given taxon is present. Row ´Total´ provides the number of sequences per taxon found in all samples. Columns starting with the prefix ´VT´ indicate specific AM fungal taxa. BD3, DG13, DG14, DG15, DG16, DG17, DG18, DG19, KEX-CG1, KIK-SS1, SK-C7, and SS-PG1 are AM

fungal taxa not included in the *MaarjAM* database (Öpik et al. 2010). Figures within each cell in the table shows the number of sequences.

Data S2. Biodiversity metrics. Column ´Habitat type" codes the position within a sampling spot into "Hedgerows", "Herbaceous crops" or "Woody crops". Columns ´D_rich´, ´D_tdiv´, ´D_func´, ´D_mntd´, ´D_med´, ´D_min´, ´D_pd´, ´D_mpd´ display the data used for Fig. 1 and Fig. S2. The prefix "D_" stands for the difference in each biodiversity metric between a sample and its closest hedgerow. Namely, cells in hedgerows´ rows should contain "zeros" for these variables: ´rich" stands for richness (i.e., the number of AM fungal virtual taxa); ´tdiv´ stands for taxonomic VT diversity (i.e., interpolated/extrapolated exponential Shannon VT diversity index); ´func´ stands for functional VT diversity (i.e., log (uncultured/ (cultured + uncultured)) taxa; ´mntd´ stands for phylogenetic VT divergence as computed by the mean nearest taxon distance; ´med´ stands for rarefied richness to the median number of VT sequences; ´min´ stands for rarefied richness to the minimum number of VT sequences; ´pd´ stands for phylogenetic richness as calculated by the phylogenetic VT diversity index; ´mpd´ stands for phylogenetic VT divergence as computed by the mean pairwise distance. Other columns are defined in the description of Data S1.

Data S3. Soil characteristics. Column ´Sample´ is defined in the column ´Sample/Hit´ of Data S1; Column ´pH´ indicates the acidity of a given soil sample; column ´Carbon´ indicates the percentage of Carbon in soil samples; OM stands for organic matter; Column ´Nitrogen´ indicates the percentage of Nitrogen in the soil samples; Column ´C:N´ provides the ratio between the columns ´Carbon´ and ´Nitrogen´. Column ´P_2O_5´provides the concentration of Olsen Phosphorus in soil samples (i.e., expressed as milligrams of phosphorus per kilogram of soil).

Data S4. Relative VT abundance. Columns ´Sample´, ´Site´, ´Sampling spot´, ´Position´, and ´Habitat type´ are defined in the description of Data S2. Columns starting with the prefix ´VT´ or ´BD3´, ´DG13´, ´DG14´, ´DG15´, ´DG16´, ´DG17´, ´DG18´, ´DG19´, ´KEX-CG1´, ´KIK-SS1´, ´SK-C7´, and ´SS-PG1´ provide the relative abundance of specific AM fungal taxa. The relative abundance of these taxa is the division of the number of sequences per taxon and sample by the total number of VT sequences of that sample.

Data S5 Representative sequences of collected samples as submitted to GenBank with accession number KFUP0000000. The detailed legend can be found in the format templates at the following link: https://submit.ncbi.nlm.nih.gov/subs/
