# Supplementary material for: Hedgerows increase the diversity and modify the composition of arbuscular mycorrhizal fungi in Mediterranean agricultural landscapes
Source: Mycorrhiza. 2022 Sep 10;32(5-6):397–407. doi: 10.1007/s00572-022-01090-5 (PMC9561024; doi:10.1007/s00572-022-01090-5)
Supplement: Supplementary file 2 — Supplementary file2 (PDF 466 KB) [file 572_2022_1090_MOESM2_ESM.pdf]

Table S1. Woody plant species in hedgerows by site. First column indicates woody plant species. Second to fifth columns are different sites.

**Species**	**Los Billares**	**El Peral**	**Fuente del Albañal**	**Vista Alegre**
Bupleurum fruticosum	X	X		
Colutea arborescens	X	X		X
Crataegus monogyna	X	X	X	X
Daphne gnidium	X			X
Dorycnium pentaphyllum		X		X
Ephedra nebrodensis	X			
Genista scorpius	X	X		
Jasminum fruticans	X	X	X	X
Juniperus oxycedrus	X	X		X
Lavandula latifolia			X	
Osiris alba			X	
Phyllirea angustifolia	X		X	X
Pistacia lentiscus			X	
Pistacia terebinthus	X	X	X	
Prunnus dulcis	X		X	X
Prunnus spinosa	X			
Quercus coccifera	X	X		X
Quercus ilex	X	X	X	X
Retama sphaerocarpa	X	X	X	X
Rhamnus alaternus	X	X	X	X
Rhamnus lycioides	X	X		X
Rosa canina	X	X	X	X
Rosmarinus officinalis			X	
Number of woody plant species	18	14	13	14
