# Supplementary material for: Hedgerows increase the diversity and modify the composition of arbuscular mycorrhizal fungi in Mediterranean agricultural landscapes
Source: Mycorrhiza. 2022 Sep 10;32(5-6):397–407. doi: 10.1007/s00572-022-01090-5 (PMC9561024; doi:10.1007/s00572-022-01090-5)
Supplement: Supplementary file 3 — Supplementary file3 (PDF 468 KB) [file 572_2022_1090_MOESM3_ESM.pdf]

Table S2 Correlations among soil parameters and biodiversity metrics. Values shown represent Kendall´s tau rank correlation coefficient (τ), with Bonferroni correction. Subscripts show the degrees of freedom of Student's *t* tests against zero; p indicates the associated probability. Significant values are highlighted in bold. Data for building this table excluded outliers. Biodiversity metrics are shown in order of appearance in the main text. OM stands for organic matter.

Biodiversity metric	pH	C (%)	$OM_{oxidable}$ (%)	OM_{total} (%)	N (%)	C:N	P_2O_5 (mg/Kg)
Richness	**τ = 0.35; t_{103} = 3.76; p = 0.02**	**τ = 0.37; t_{103} = 4.01; p = 0.01**	**τ = 0.33; t_{103} = 3.50; p = 0.04**	**τ = 0.33; t_{103} = 3.58; p = 0.03**	**τ = 0.35; t_{103} = 3.82; p = 0.01**	τ = -0.03; t_{103} = -0.33; p = 1.00	τ = -0.13; t_{103} = -1.40; p = 1.00
Taxonomic diversity	τ = 0.18; t_{103} = 1.95; p = 1.00	τ = 0.22; t_{103} = 2.29; p = 1.00	τ = 0.18; t_{103} = 1.91; p = 1.00	τ = 0.19; t_{103} = 1.92; p = 1.00	τ = 0.22; t_{103} = 2.30; p = 1.00	τ = -0.15; t_{103} = -1.54; p = 1.00	τ = -0.08; t_{103} = -0.84; p = 1.00
Functional diversity	τ = 0.08; t_{103} = 0.79; p = 1.00	τ = 0.20; t_{103} = 2.06; p = 1.00	τ = 0.19; t_{103} = 1.94; p = 1.00	τ = 0.18; t_{103} = 1.88; p = 1.00	τ = 0.18; t_{103} = 1.86; p = 1.00	τ = -0.09; t_{103} = -0.90; p = 1.00	τ = -0.15; t_{103} = -1.54; p = 1.00
Phylogenetic divergence (mntd)	**τ = -0.51; t_{103} = -6.01; p < 0.01**	**τ = -0.29; t_{103} = -3.11; p = 0.01**	**τ = -0.30; t_{103} = -3.18; p < 0.01**	**τ = -0.31; t_{103} = -3.29; p < 0.01**	τ = -0.32; t_{103} =- 3.40; p = 0.07	τ = -0.11; t_{103} = -1.16; p = 1.00	τ = 0.21; t_{103} = 2.18; p = 1.00
Rarefied richness to the median number of sequences	τ = 0.18; t_{103} = 1.83; p = 1.00	τ = 0.20; t_{103} = 2.04; p = 1.00	τ = 0.17; t_{103} = 1.78; p = 1.00	τ = 0.18; t_{103} = 1.85; p = 1.00	τ = 0.18; t_{103} = 1.89; p = 1.00	τ = -0.05; t_{103} = -0.50; p = 1.00	τ = -0.13; t_{103} = -1.30; p = 1.00
Rarefied richness to the minimum number of sequences	τ = 0.25; t_{103} = 2.64; p = 0.54	τ = 0.33; t_{103} = 3.60; p = 0.04	τ = 0.29; t_{103} = 3.05; p = 1.00	τ = 0.30; t_{103} = 3.15; p = 0.12	τ = 0.32; t_{103} = 3.40; p = 0.05	τ = -0.07; t_{103} = -0.77; p = 1.00	τ = -0.07; t_{103} = -0.69; p = 1.00
Faith's phylogenetic diversity index (pd)	τ = 0.18; t_{103} = 1.86; p = 1.00	τ = 0.17; t_{103} = 1.73; p = 1.00	τ = 0.12; t_{103} = 1.19; p = 1.00	τ = 0.12; t_{103} = 1.23; p = 1.00	τ = 0.17; t_{103} = 1.78; p = 1.00	τ = -0.14; t_{103} = -1.46; p = 1.00	τ = -0.05; t_{103} = -0.49; p = 1.00
Mean nearest taxon distance (mpd)	**τ = -0.39; t_{103} = -4.31; p < 0.01**	τ = -0.35; t_{103} = -3.81; p = 0.13	τ = -0.37; t_{103} = -4.06; p = 0.11	τ = -0.38; t_{103} = -4.12; p = 0.08	τ = -0.31; t_{103} = -3.31; p = 0.57	τ = -0.09; t_{103} = -0.97; p = 1.00	τ = 0.18; t_{103} = 1.85; p = 1.00
