# Supplementary material for: Hedgerows increase the diversity and modify the composition of arbuscular mycorrhizal fungi in Mediterranean agricultural landscapes
Source: Mycorrhiza. 2022 Sep 10;32(5-6):397–407. doi: 10.1007/s00572-022-01090-5 (PMC9561024; doi:10.1007/s00572-022-01090-5)
Supplement: Supplementary file 4 — Supplementary file4 (PDF 378 KB) [file 572_2022_1090_MOESM4_ESM.pdf]

Table S3. Complete PERMANOVA table. Habitat type codes for “Hedgerows”, “Herbaceous crops", and “Woody crops”. “Site” codes for “El Peral”, “Vista Alegre”, “Fuente del Albañal”, and “Los Billares”; r indicates the Pearson correlation coefficient. F is the threshold statistic; p stands for p-value.

Variable	Degrees of freedom	Sum of squares	r	F	p
Habitat type	2	2.46	0.07	45.00	< 0.01
Site	3	3.54	0.10	47.00	< 0.01
Residual	109	28.58	0.83		
Total	114	34.60	1.00		
