# Supplementary material for: Hedgerows increase the diversity and modify the composition of arbuscular mycorrhizal fungi in Mediterranean agricultural landscapes
Source: Mycorrhiza. 2022 Sep 10;32(5-6):397–407. doi: 10.1007/s00572-022-01090-5 (PMC9561024; doi:10.1007/s00572-022-01090-5)
Supplement: Supplementary file 5 — Supplementary file5 (PDF 462 KB) [file 572_2022_1090_MOESM5_ESM.pdf]

Table S4. Indicator taxa by farmland habitat type. Only indicator values higher than 0.25 are shown.

**Virtual Taxa**	**Morphospecies**	**Main AM fungal host**	**Indicator Value**	**Probability**
VT380	*Diversispora* Torrecillas 12b Div2	Hedgerows	0.62	0.001
VT105	*Glomus* Torrecillas 13 Glo G3	Hedgerows	0.53	0.001
VT342	*Glomus* Torrecillas 12b Glo G18	Hedgerows	0.47	0.001
VT143	*Glomus sp.*	Hedgerows	0.45	0.001
VT54	*Diversispora sp.*	Hedgerows	0.38	0.011
VT295	*Glomus sp.*	Hedgerows	0.34	0.009
VT388	*Glomus* Alguacil 09b	Hedgerows	0.33	0.034
VT61	*Diversispora sp.*	Hedgerows	0.30	0.002
VT193	*Claroideoglomus* Glo G8	Woody crops	0.49	0.004
VT357	*Claroideoglomus* Alguacil 12b GLO G3	Woody crops	0.48	0.001
VT113	*Glomus sp.*	Woody crops	0.34	0.006
VT387	*Glomus* Alguacil 09b Glo G14	Woody crops	0.27	0.002
VT444	*Paraglomus* IH1	Herbaceous crops	0.67	0.001
VT245	*Archaeospora sp.*	Herbaceous crops	0.62	0.001
VT05	*Archaeospora sp.*	Herbaceous crops	0.43	0.001
VT284	*Pacispora scintillans*	Herbaceous crops	0.38	0.015
