# Supplementary material for: Hedgerows increase the diversity and modify the composition of arbuscular mycorrhizal fungi in Mediterranean agricultural landscapes
Source: Mycorrhiza. 2022 Sep 10;32(5-6):397–407. doi: 10.1007/s00572-022-01090-5 (PMC9561024; doi:10.1007/s00572-022-01090-5)
Supplement: Supplementary file 6 — Supplementary file6 (PDF 508 KB) [file 572_2022_1090_MOESM6_ESM.pdf]

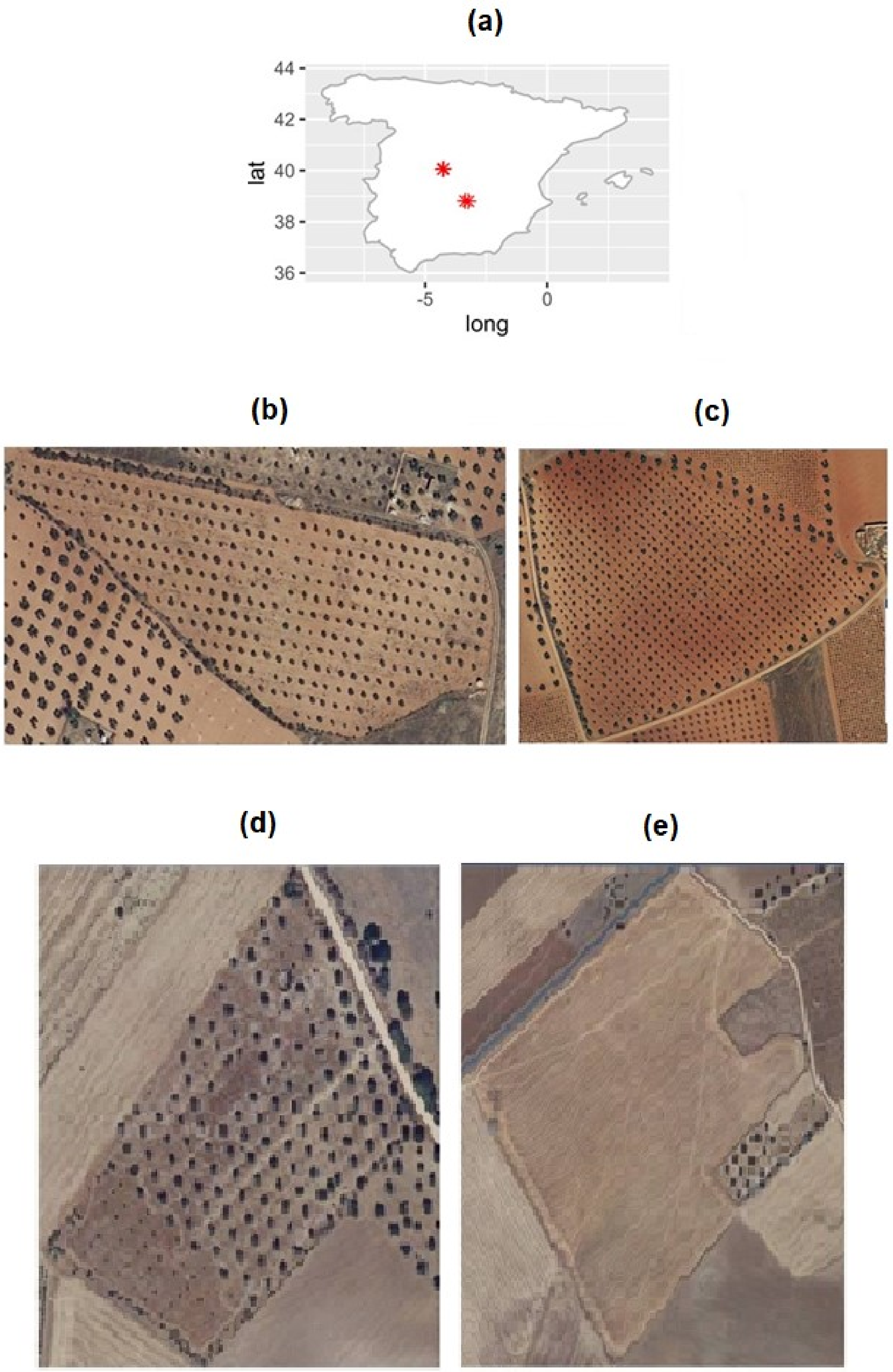

Figure S1. Situation map (a) and aerial photographs of the studied fields taken from SIGPAC (https://sigpac.mapama.gob.es/fega/visor/). "El Peral" (b; 38º48´N, 3º21´W; 1.76 hectares), “Vista Alegre" (c; 38º48´N, 3º 13´W; 4.55 hectares), "Fuente del Albañal" (d; 40º3´N, 4º 17`W; 2.65 hectares), and "Los Billares" (e; 40º 1´N, 4º 14´W; 12.66 hectares) sites.
