# Supplementary material for: Hedgerows increase the diversity and modify the composition of arbuscular mycorrhizal fungi in Mediterranean agricultural landscapes
Source: Mycorrhiza. 2022 Sep 10;32(5-6):397–407. doi: 10.1007/s00572-022-01090-5 (PMC9561024; doi:10.1007/s00572-022-01090-5)
Supplement: Supplementary file 7 — Supplementary file7 (PDF 590 KB) [file 572_2022_1090_MOESM7_ESM.pdf]

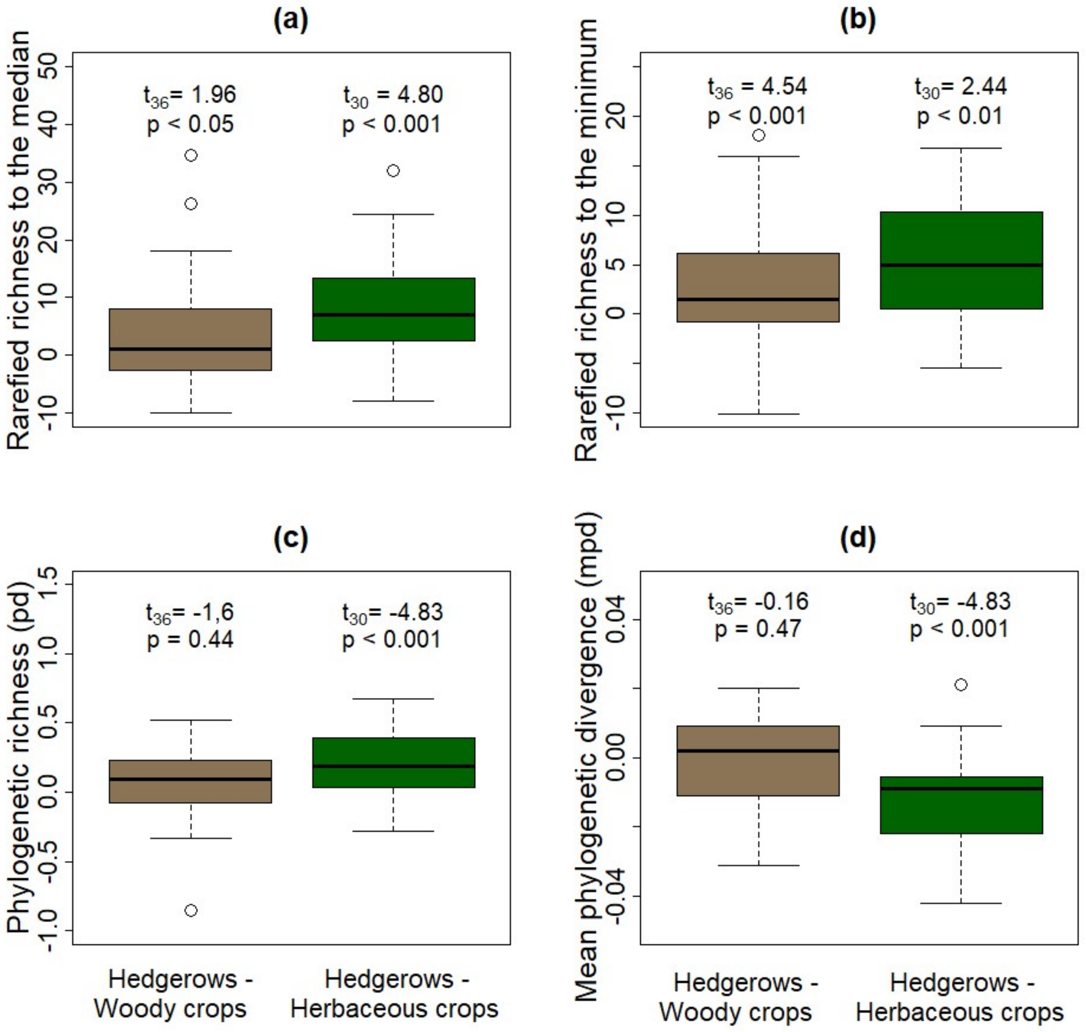

Figure S2 Rarefied richness to the median number of VT sequences (a), rarefied richness to the minimum number of VT sequences (b), phylogenetic VT richness (i.e., phylogenetic diversity index, c) and phylogenetic VT divergence (i.e., mean pairwise distance, d). Ordinates show the differences (not the actual values) between hedgerow and crop samples in each sampling spot (the hedgerow sample arbuscular mycorrhizal fungal biodiversity metric in sampling spot X minus the crop sample arbuscular mycorrhizal fungal biodiversity metric in sampling spot X). These differences were tested based on one-tailed Student T tests for positive effects of hedgerows. The thick black horizontal line displays the median difference of a given biodiversity metric. Boxes are constrained by interquartile range. Whiskers are limited by 1.5 times the interquartile range beyond the first and third quartiles. Open circles represent possible outliers. Text below boxes indicates the Student *t*-statistic value, subscripts show the degrees of freedom, and p represent the associated probability.
